# Ectopic Expression of MADS-Box Transcription Factor *VvAGL12* from Grape Promotes Early Flowering, Plant Growth, and Production by Regulating Cell-Wall Architecture in *Arabidopsis*

**DOI:** 10.3390/genes14112078

**Published:** 2023-11-15

**Authors:** Tingting Mao, Xueting Wang, Hongsheng Gao, Zijian Gong, Ruichao Liu, Ning Jiang, Yaru Zhang, Hongxia Zhang, Xiaotong Guo, Chunyan Yu

**Affiliations:** 1 The Engineering Research Institute of Agriculture and Forestry, Ludong University, 186 Hongqizhong Road, Yantai 264025, China; 2 College of Agriculture, Ludong University, 186 Hongqizhong Road, Yantai 264025, China; 3 Shandong Institute of Sericulture, Shandong Academy of Agricultural Sciences, 21 Zhichubei Road, Yantai 264001, China

**Keywords:** grape, *VvAGL12*, plant growth, cell wall

## Abstract

The MADS-box family, a substantial group of plant transcription factors, crucially regulates plant growth and development. Although the functions of AGL12-like subgroups have been elucidated in *Arabidopsis*, rice, and walnut, their roles in grapes remain unexplored. In this study, we isolated *VvAGL12*, a member of the grape MADS-box group, and investigated its impact on plant growth and biomass production. *VvAGL12* was found to localize in the nucleus and exhibit expression in both vegetative and reproductive organs. We introduced *VvAGL12* into *Arabidopsis thaliana* ecotype Columbia-0 and an *agl12* mutant. The resulting phenotypes in the *agl12* mutant, complementary line, and overexpressed line underscored *VvAGL12’*s ability to promote early flowering, augment plant growth, and enhance production. This was evident from the improved fresh weight, root length, plant height, and seed production, as well as the reduced flowering time. Subsequent transcriptome analysis revealed significant alterations in the expression of genes associated with cell-wall modification and flowering in the transgenic plants. In summary, the findings highlight *VvAGL12′*s pivotal role in the regulation of flowering timing, overall plant growth, and development. This study offers valuable insights, serving as a reference for understanding the influence of the *VvAGL12* gene in other plant species and addressing yield-related challenges.

## 1. Introduction

MADS-box transcription factors represent a crucial family of transcription factors within eukaryotes and have garnered extensive attention and research in the realms of plants, animals, and fungi. This family is characterized by a highly conserved DNA-binding domain and can be classified into two primary categories: Type I and Type II [1]. Although Type I MADS-box factors have not been extensively investigated, their Type II counterparts, also known as MIKC MADS-box factors, have been extensively explored. MIKC MADS-box factors encompass four distinct domains: the MADS box, intervening region, keratin box, and C-terminal domain. Furthermore, this category can be further subdivided into MIKC^C^ and MIKC* based on the presence or absence of keratin boxes. Among these, the MIKC^C^ MADS-box factors have emerged as the most extensively researched, exhibiting a well-defined mechanism. MIKC^C^ MADS-box factors can be classified into 12 subfamilies: AG-like, AGL2-like, AGL6-like, AGL12-like, AGL15-like, AGL17-like, DEF/GLO-like, FLC-like, GGM13-like, SQUA-like, STMADS11-like, and TM3-like [2].

The MADS-box family has a significant influence on both plant and fungal systems [3]. Its pivotal role extends to various aspects of reproductive growth, encompassing floral organ development, ovule development, and seed oil synthesis [4,5,6]. Additionally, this family contributes to the regulation of plant root development and the overall plant configuration [7]. Extensive research has shed light on MADS-box members in various species. For instance, in grape plants, the complex involving VvMADS39, AG, and AGL11 serves as a critical regulator of flower and ovule development, ultimately affecting the formation of seedless fruits [8]. *Medicago truncatula* relies on *MtFULc* to govern inflorescence development, working in coordination with *MtTFL1* and *MtPIM* [9]. In soybean, *GmFULa* improves vegetative growth and boosts soybean yield by positively regulating sucrose synthases (*SUSs*) and sucrose transporters (*SUTs*), without altering flowering time or maturity [10]. In *Brassica napus*, the presence of *Bna.AP.A02*, an *AP1* ortholog mutant, induces notable changes in flower morphology, plant architecture, and seed yield components within the oilseed rape plant [11]. Beyond its roles in reproduction and development, the MADS-box family also proves crucial in responding to abiotic stresses, such as hormonal and salt stress, drought stress, and low-temperature stress [12,13,14].

Functional attributes of the AGL12-like subgroup have only been documented in *Arabidopsis thaliana*, rice (*Oryza sativa*), and walnut (*Juglans* sp.). In *Arabidopsis*, *AtAGL12* is associated with root meristem cell proliferation, flowering transition, and cell cycle regulation. Furthermore, *AtAGL12* affects somatic embryo germination rates and root configurations in walnut [15,16,17]. In rice, plants overexpressing *OsMADS26* exhibit stress-related phenotypes, including chlorosis, cell death, pigment accumulation, and growth defects in the roots and buds. Conversely, downregulation of *OsMADS26* enhances resistance to pathogenic bacteria and drought stress [18]. However, the biological functions and regulatory mechanisms of AGL12 in grapes remain unclear.

Grapes (*Vitis vinifera*) are globally significant, serving not only as a valuable economic commodity with high nutritional value but also as a renowned medicinal resource [19]. Although the *AGL12* genes in rice, *Arabidopsis*, and walnut have been extensively characterized, the biological functions of *AGL12* in grapes remain unreported and warrant further investigation. In this study, we isolated the AGL12-like gene (*VvAGL12*) from the “Pinot Noir” variety of *Vitis vinifera* and conducted investigations into its potential roles in regulating flowering time, plant growth, and productivity in *Arabidopsis*. The findings revealed that *VvAGL12* played a pivotal role as a key regulator influencing both vegetative and reproductive growth in plants through the expansion and elongation of cell walls in *Arabidopsis*. This study provides valuable insights into the biological functions and regulatory mechanisms of *VvAGL12* in grapes.

## 2. Materials and Methods

### 2.1. Plant Materials and Growth Conditions

In the experiment, four *Arabidopsis* varieties were utilized, comprising the wild-type (WT), mutant (*agl12*), complementary line (*Vv12:cs*), and overexpression line (*Vv12:*col). The *Arabidopsis* wild-type variety Columbia-0 (Col-0) was maintained in our lab, and the mutant variety was procured from the Arabidopsis Biological Resources Center (ABRC).

Firstly, sterile cultivation and vernalization of *Arabidopsis* were initiated on the MS medium. Once the seedlings reached the four-leaf stage, they were transplanted into a soil mixture consisting of nutrient soil and vermiculite at a 2:1 ratio. To promote *Arabidopsis* growth, environmental conditions were set in a culture room at 23 °C, maintaining an air humidity level of 70–80%, and employing a 16 h light/8 h dark photoperiod.

Tobacco plants (*Nicotiana benthamiana*) were cultivated under conditions of 14 h of light and 10 h of dark, at a temperature of 25 °C and a relative humidity of 70%. These plants were incubated for approximately 4–5 weeks before the subcellular localization experiment was started.

### 2.2. Homologous Alignment and Evolutionary Tree Analysis

The amino acid sequences of the AGL12 orthologs were retrieved from Phytozome13 (https://phytozome-next.jgi.doe.gov/, accessed on 11 January 2021). The homologous alignment of AGL12 sequences was conducted using the DNAMAN V6 software [20]. Subsequently, an AGL12 phylogenetic tree was constructed using the MEGA-X software, employing the maximum likelihood (ML) method with 1000 bootstraps [21].

### 2.3. Cloning and Vector Construction of Grape VvAGL12 Gene

To clone the *VvAGL12* gene, specific primers were designed based on the cDNA of the sequenced grape variety Pinot Noir. RNA was extracted from a mixture of root and leaf tissues of Pinot Noir, and cDNA templates were synthesized through reverse transcription. Subsequently, the target gene *VvAGL12* was cloned and integrated into the pCAMBIA2301 vector following double enzyme digestion (*Bam*H I and *Sal* I). This resulted in the creation of an overexpression vector, pCAMBIA2301: 35S: VvAGL12, for *Arabidopsis* transformation. Concurrently, the target gene was ligated into the pCAMBIA1300: 35S: YFP vector to construct a recombinant vector, YFP:: VvAGL12, for subcellular localization experiments. The combined vectors were transformed into *Escherichia coli* for PCR validation and sequencing. Upon successful verification by PCR and sequencing, the recombinant plasmid was transformed into *Agrobacterium tumefaciens* GV3101 for further investigation.

### 2.4. Subcellular Localization

Agrobacterium tumefaciens carrying the recombinant plasmids YFP:: VvAGL12 and YFP were used to infect epidermal cells on the abaxial surface of tobacco plant leaves. Following this, the tobacco plants underwent a 12 h period of darkness, followed by 2 d of incubation under light conditions. Subsequently, marked sections of the tobacco leaves were excised, and VvAGL12 subcellular localization was visualized using a laser confocal microscope [22].

### 2.5. Transformation and Phenotypic Identification of VvAGL12 Transgenic Arabidopsis

Precisely identified Agrobacterium tumefaciens carrying the pCAMBIA2301: 35S: VvAGL12 vector was employed to infect the inflorescences of *Arabidopsis* (Col-0 and *agl12* mutant). Subsequently, the transgenic seeds from the T_0_ generation were collected. Following kanamycin resistance screening and PCR verification, homozygous *VvAGL12 Arabidopsis* from the T_3_ generation was obtained for subsequent experiments.

Following disinfection and vernalization, seeds from the wild-type (Col-0), mutant (*agl12*), complementary mutant (*Vv12*:*cs*), and overexpressed *Arabidopsis* (*Vv12:*col) strains were sown and cultivated on the MS medium. The primary root length of *Arabidopsis* was then observed and measured 7 d after planting. Concurrently, *Arabidopsis* seedlings with four developed leaves were transplanted into nutrient soil (vermiculite: organic soil = 2:1). The experiments were designed to be completely randomized. Various growth parameters including growth trajectory, bolting period, flowering period, rosette leaf count, and yield-related traits were monitored across different growth stages.

### 2.6. Transcriptome Sequencing

*Arabidopsis* seedlings from the WT, *agl12*, and *Vv12:*col lines were subjected to transcriptome sequencing (RNA-Seq). Following seed sterilization and vernalization, the *Arabidopsis* seeds were sown on the same MS medium. After a 10 d growth period, 10 whole plants were selected from each line for RNA extraction. The concentration and quality of RNA in the selected samples were assessed using an Agilent 2100 bioanalyzer [23]. Subsequently, a sequence library was generated using an Illumina NovaSeq 6000 platform (Illumina, San Diego, CA, USA) [24]. Raw read count data were mapped to the *Arabidopsis* Col-0 genome (www.Arabidopsis.org) in the TAIR10 database. Expression levels of each gene were represented as FPKM values. Differentially expressed genes (DEGs) were filtered using a false discovery rate (FDR) < 0.05 and a log_2_ (fold) > 1 threshold [25]. To obtain gene annotation information and enrich all genes, the clusterProfiler V4.0 software package was used for the GO/KEGG analysis [26]. Subsequently, the ggplot2 software package was utilized to visualize the results.

### 2.7. RNA Extraction and Real-Time Quantitative PCR Analysis

To elucidate the tissue-specific expression pattern of the *VvAGL12* gene, various tissues, including young leaves, old leaves, tendrils, young fruits, inflorescences, stems, and root tissues, were sampled from the sequenced grape variety Pinot Noir under natural conditions. Subsequently, RNA was extracted from each tissue sample to analyze the specific expression levels of the *VvAGL12* gene. All RT-qPCR reactions were performed in triplicates using an ABI StepOne circulator (Thermo Fisher, Singapore). Target gene expression values were normalized based on the expression of the reference gene actin for grapes [27].

To verify the differential genes detected by RNA-Seq, RNA was extracted from *Arabidopsis* seedlings wild-type (WT), mutant (*agl12*), and overexpressed line (*VvAGL12*:col), and cDNA was obtained using a reverse transcription kit as a template for the validation of the differential genes detected by RNA-Seq. The actin gene of *Arabidopsis* was used as an internal reference and was calculated using the 2^−ΔΔCT^ method [28].

### 2.8. Statistical Analysis

All experiments were repeated three times, and data analyses were conducted using SPSS 22. Results were presented as mean ± standard error (SE). Data analysis involved one-way ANOVA, followed by Duncan’s multiple range test to assess statistical significance (* *p* < 0.05 and ** *p* < 0.01). Graphical representations were generated using GraphPad Prism 8.

## 3. Results

### 3.1. Identification and Expression Pattern Analysis of the AGL12 Gene in Grapevine

**Figure 1:**
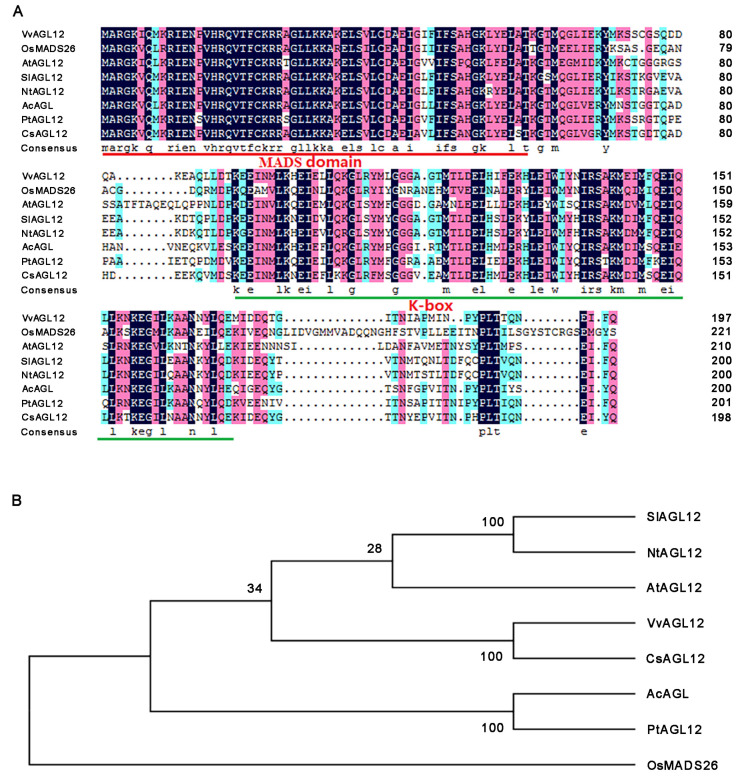
Multiple sequence alignment and evolutionary tree analysis of VvAGL12 and other plant AGL12 proteins. (**A**) Sequence alignment of VvAGL12 and homologous AGL12 proteins from various plants. The protein accession numbers were as follows: VvAGL12 (GenBank: XP_002278239.1), OsMADS26 (GenBank: NP_001390263.1), AtAGL12 (GenBank: NP_565022), SlAGL12 (GenBank: NP_001233764.2), NtAGL12 (GenBank: NP_001312055.11), AcAGL12 (GenBank: PSS00173.1), PtAGL12 (GenBank: XP_006376144.2), and CsAGL12 (GenBank: XP_028060316.1). Conserved domains were highlighted in red and green lines. (**B**) Phylogenetic tree of VvAGL12 with homologous AGL12 proteins from other plants.

The *VvAGL12* gene was identified within the Pinot Noir grapevine genome, with an ORF length of 597 bp encoding 198 amino acids. Furthermore, the genomic location of the *VvAGL12* gene was determined to be on chromosome 18. Domain analysis revealed the presence of both conserved MADS-box and K-box domains in VvAGL12 (Figure 1A), confirming its classification within the MADS family. Phylogenetic tree analysis demonstrated that the VvAGL12 protein from grapes exhibited highest similarity to the CsAGL12 protein from the tea tree and lowest similarity to the OsMADS26 protein from the AGL12-clade in rice (Figure 1B).

**Figure 2:**
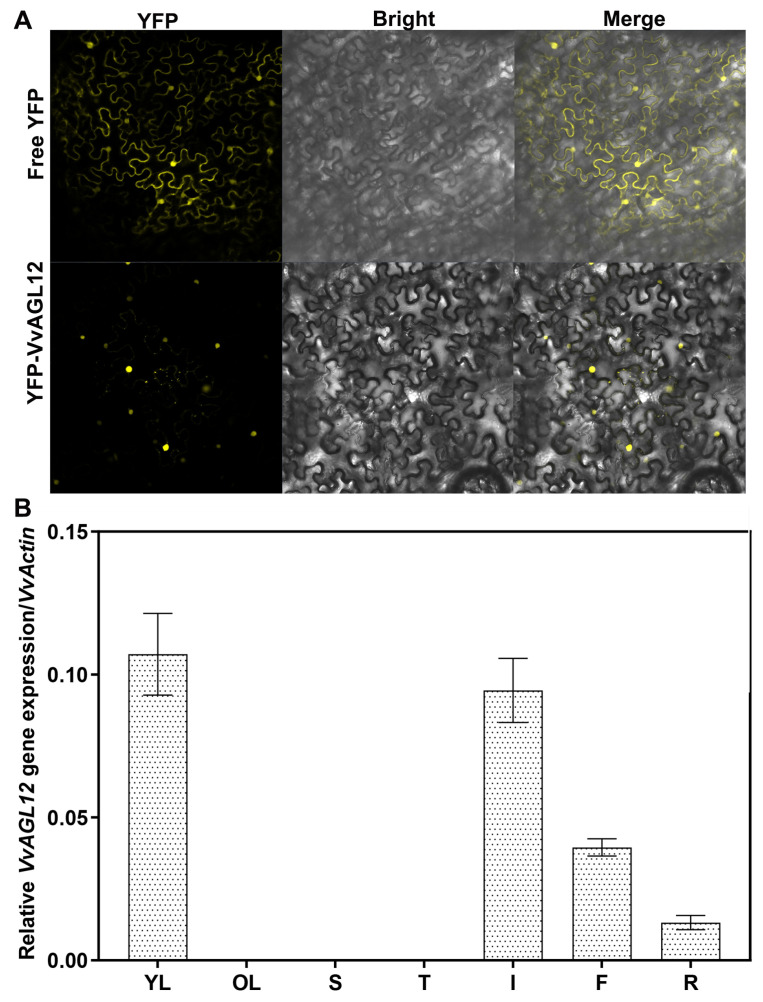
Subcellular localization and tissue-specific expression analysis of VvAGL12 protein. (**A**) Subcellular localization of VvAGL12 protein. (**B**) Tissue-specific expression analysis of *VvAGL12* in various tissues of Pinot Noir plants. YL: young leaves; OL: old leaves; S: stem; T: tendrils; I: inflorescence; F: small fruit; R: root.

To determine the localization of VvAGL12, a fusion construct of VvAGL12 with the YFP C-terminus (YFP:: VvAGL12) was generated and introduced into leaf cells. The YFP fluorescence signal was observed using a laser confocal microscope. The results revealed the predominant expression of the YFP:: VvAGL12 fusion protein within the nucleus (Figure 2A), indicating that VvAGL12 functions as a nuclear transcription factor.

To delve deeper into the expression profile of *VvAGL12*, a tissue expression pattern analysis was performed using qRT-PCR. As depicted in Figure 2B, *VvAGL12* exhibited elevated expression levels in young leaves and inflorescences, followed by detectable expression in small fruits and roots (Figure 2B). These findings imply a potential pivotal role for the *VvAGL12* gene in plant growth and flowering.

### 3.2. Ectopic Expression of VvAGL12 Enhanced Primary Root Development and Flowering Time in Transgenic Arabidopsis

To investigate the biological role of *VvAGL12* in plant growth and development, Agrobacterium tumefaciens carrying the 35S:: VvAGL12 construct was separately transformed into WT and mutant *Arabidopsis* plants to generate overexpression and complementation lines. Following selection on the appropriate medium and DNA verification, a total of eight positive transgenic and 13 complementation plants were identified (Figure S1). Subsequently, lines with the highest *VvAGL12* expression levels were selected for further analysis using qRT-PCR. Specifically, *Vv12:*col represents the transgenic line with the highest *VvAGL12* expression level, whereas *Vv12:cs* denotes the complementation line with the highest expression.

**Figure 3:**
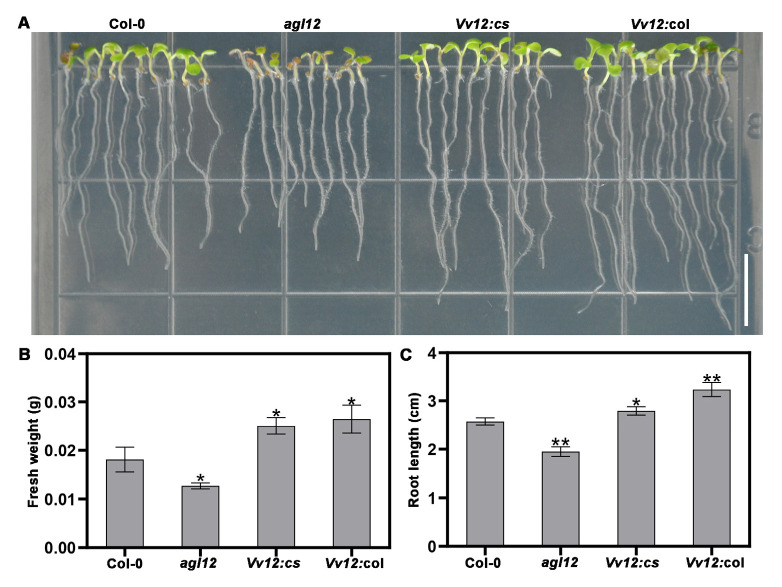
*VvAGL12* promoted elongation of the main roots and the growth of *Arabidopsis* seedlings. (**A**) Phenotypes of wild-type, mutant, and transgenic *Arabidopsis* seedlings grown vertically on MS plates for 9 d. Scale bar = 1 cm. (**B**) Fresh weight and (**C**) primary root length calculated for four lines: Col-0 (wild-type *Arabidopsis*), *agl12* (mutant of *AGL12*), *Vv12:cs* (*VvAGL12* complementary *Arabidopsis* mutant *agl12*), and *Vv12:*col (overexpressed *VvAGL12* in wild-type). Asterisks indicate significant differences between the wild-type (Col-0) and other three lines (* *p* < 0.05; ** *p* < 0.01).

Four lines (Col-0, *agl12*, *Vv12*:*cs*, and *Vv12:*col) were co-cultivated in MS dishes to observe their phenotypes (Figure 3A). After 7 d, it was evident that the *Vv12:*col seedlings had a greater fresh weight than the other three lines (Figure 3B). Additionally, the primary root length of the *Vv12:*col seedlings after 7 d exceeded that of the other three lines (Figure 3C). Notably, among the four lines, *agl12* exhibited the shortest primary root length, which was consistent with its lower fresh weight. Importantly, overexpression of *VvAGL12* in *agl12* plants successfully rescued these phenotypic differences (Figure 3B). These results strongly suggest that the *VvAGL12* gene plays a role in primary root development and growth.

**Figure 4:**
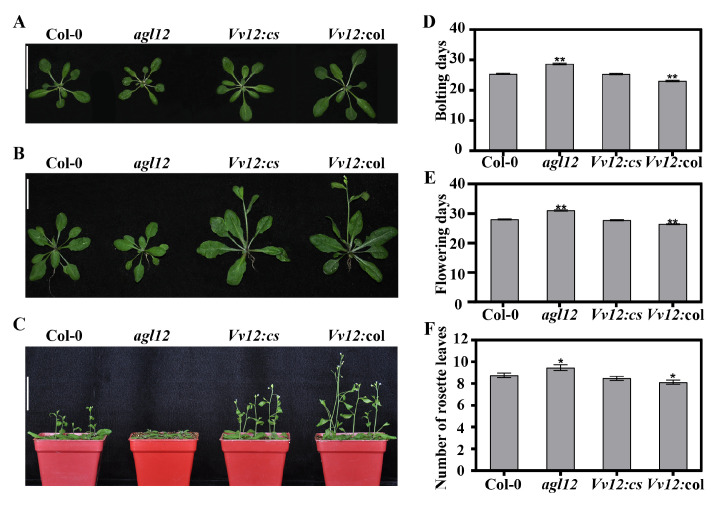
Overexpression of *VvAGL12* promoted flowering in *Arabidopsis thaliana*. (**A**) Representative images of 20-day-old Col-0 (wild-type), *agl12* (mutant), and *VvAGL12* transgenic *Arabidopsis* lines. Compared to wild-type Col-0, *Vv12:*col had a larger shoot size, whereas *agl12* had a smaller shoot size. Scale bar = 1.5 cm. (**B**) Overexpression of *VvAGL12* led to early bolting in *Arabidopsis*. Representative images of 25-day-old Col-0 (wild-type), *agl12* (mutant), and *VvAGL12* transgenic *Arabidopsis* lines were captured. *Vv12:*col and *Vv12:cs* exhibited early bolting, whereas Col-0 and *agl12* did not. Scale bar = 1.5 cm. (**C**) Overexpression of *VvAGL12* showed early flowering in *Arabidopsis*. The 30-day-old Col-0 (wild-type), *agl12* (mutant), and *VvAGL12* transgenic *Arabidopsis* lines were photographed. Scale bar = 5 cm. (**D**) Days to bolting of Col-0 (wild-type), *agl12* (mutant), and *VvAGL12* transgenic *Arabidopsis* lines. (**E**) Days to flowering in Col-0 (wild-type), *agl12* (mutant), and *VvAGL12* transgenic *Arabidopsis* lines. (**F**) Number of rosette leaves in Col-0 (wild-type), *agl12* (mutant), and *VvAGL12* transgenic *Arabidopsis* lines. Col-0 was a wild-type *Arabidopsis*, *agl12* was a mutant of *AGL12*, *Vv12:cs* was the *VvAGL12* complementary *Arabidopsis* mutant *agl12*, and *Vv12:*col was overexpressed *VvAGL12* in the wild-type. Asterisks indicate significant differences between the wild-type (Col-0) and other three lines (* *p* < 0.05; ** *p* < 0.01).

Previous studies have highlighted the association between *AGL12* genes and flowering time regulation in plants. The expression pattern of *VvAGL12* indicated its pronounced presence in inflorescences, suggesting its potential role in flowering. To validate this hypothesis, flowering-related traits were assessed (Figure 4). Notably, the flowering and bolting time in *Vv12:*col occurred significantly earlier than in the wild-type, whereas the mutant exhibited significantly delayed flowering and bolting compared to the WT (Figure 4B–E). Concurrently, the number of rosette leaves during flowering in *Vv12:*col was significantly lower than that in the wild-type, whereas the mutant displayed a significantly higher number of rosette leaves than the wild-type during flowering. The complement lines showed no significant differences compared with the wild-type (Figure 4F). These results revealed that heterologous expression of *VvAGL12* could promote flowering in *Arabidopsis*.

### 3.3. Ectopic Expression of VvAGL12 Improved the Biomass in Transgenic Arabidopsis

**Figure 5:**
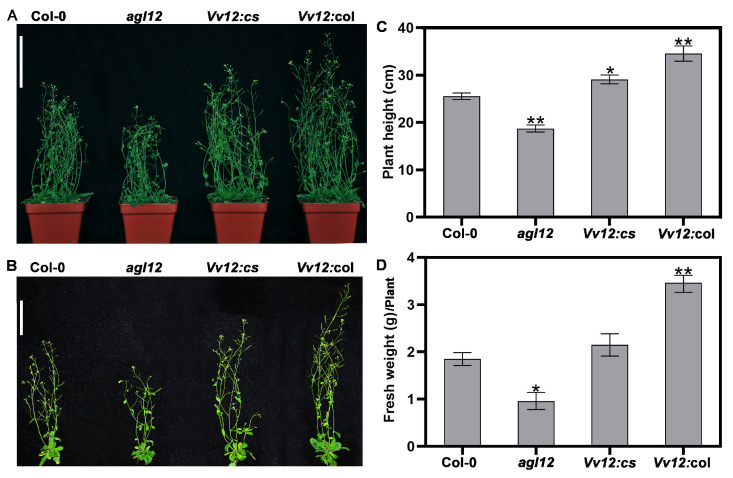
Overexpression of *VvAGL12* increased plant height and fresh weight in transgenic *Arabidopsis* during the pod stage. Plants were observed for 28 days after culturing in the soil. The phenotypes of the four lines were photographed (**A**,**B**). (**A**) Scale bar = 9 cm. (**B**) Scale bar = 6 cm. A total of 10 *Arabidopsis* plants of each variety were selected for analysis of (**C**) plant height and (**D**) fresh weight. Col-0 was a wild-type *Arabidopsis*, *agl12* was a mutant of *AGL12*, *Vv12:cs* was the *VvAGL12* complementary *Arabidopsis* mutant *agl12*, and *Vv12:*col was overexpressed *VvAGL12* in the wild-type. Asterisks indicate significant differences between the wild-type (Col-0) and the other three lines (* *p* < 0.05; ** *p* < 0.01).

Under optimal nutritional conditions, the growth advantage of the transgenic *VvAGL12* varieties was consistently maintained even after 28 d of growth (Figure 4 and Figure S2). Overexpression of *VvAGL12* in transgenic *Arabidopsis* (*Vv12:*col) resulted in larger leaves and increased the overall plant size. As the plants initiated the development of flower buds, the height and biomass measurements further highlighted these differences. Specifically, *Vv12:*col exhibited greater plant height and biomass than wild-type plants, whereas the *agl12* mutant displayed reduced plant height and biomass. Importantly, overexpression of *VvAGL12* in the *agl12* mutant successfully rescued these phenotypic differences (Figure 5). These findings affirm that the overexpression of *VvAGL12* enhances plant vegetative growth.

**Figure 6:**
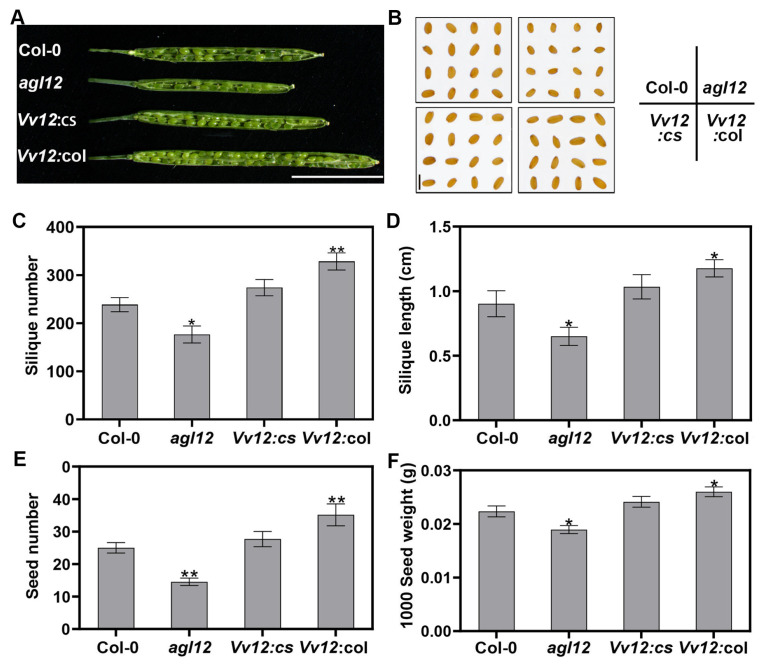
Overexpression of *VvAGL12* enhanced *Arabidopsis* seed yield. (**A**) Silique phenotypes of the four lines (Col-0, *agl12*, *Vv12:cs*, and *Vv12:*col). Overexpression of *VvAGL12* enhanced the (**B**) seed size, (**C**) silique number, (**D**) silique length, (**E**) seed number per silique, and (**F**) seed weight in *Arabidopsis*. (**A**) Scale bar = 4 mm. (**B**) Scale bar = 1 mm. Col-0 was a wild-type *Arabidopsis*, *agl12* was a mutant of *AGL12*, *Vv12:cs* was the *VvAGL12* complementary *Arabidopsis* mutant *agl12*, and *Vv12:*col was overexpressed *VvAGL12* in the wild-type. Asterisks indicate significant differences between the wild-type and the other three lines (* *p* < 0.05; ** *p* < 0.01).

Simultaneously, we examined yield-related traits pertaining to siliques and seeds. As depicted in Figure 6, loss of function of *AGL12* resulted in a more pronounced reduction in both silique number and silique length compared to WT (Figure 6C,D). Overexpression of the *VvAGL12* gene in the *agl12* mutant successfully restored the phenotype, aligning it closely with that of the WT. However, overexpression of the *VvAGL12* gene in the WT resulted in a substantially higher number and longer siliques than those in the wild-type. Given the notable alteration in silique length induced by the *VvAGL12* gene, we further analyzed the number of seeds per silique. Each silique of *Vv12:*col contained approximately 35 seeds, which was significantly higher than that in wild-type plants (approximately 25 seeds). Conversely, the number of seeds in each silique of the *agl12* mutant was approximately 14, which was significantly lower than that of the wild-type, and there were no significant differences between *Vv12:cs* and the wild-type. Additionally, *VvAGL12* transgenic plants (*Vv12:*col) exhibited larger seeds than WT plants (Figure 6E,F). These results underscore the pivotal role of *VvAGL12* in silique and seed development, contributing to increased plant yield.

### 3.4. Ectopic Expression of VvAGL12 Enhanced the Expression Level of Cell-Wall-Related Genes in Arabidopsis

**Figure 7:**
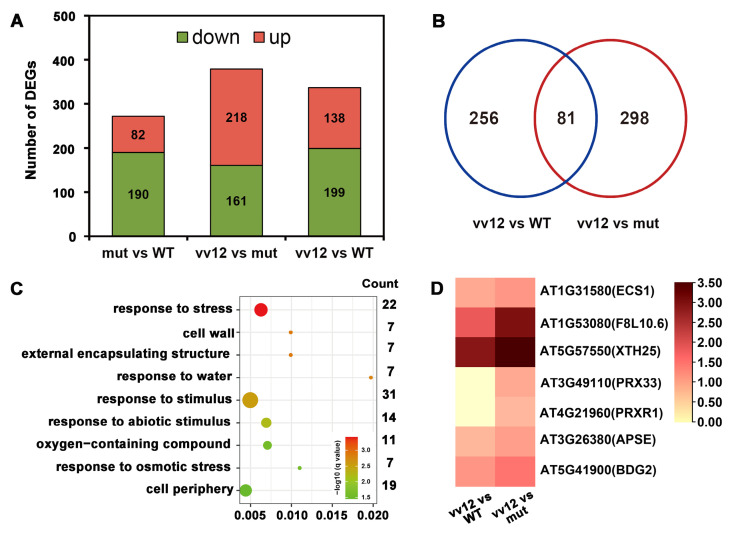
Transcriptome analysis of *Arabidopsis* wild-type, mutant, and overexpressed plants (*Vv12:*col). For each line, 10-day-old seedlings were collected for sequencing. Three comparisons (mutant vs WT, *VvAGL12* overexpressing plants (vv12) vs WT, and *VvAGL12* overexpressing plants (vv12) vs mutant) were conducted to filter the different genes. (**A**) Number of differentially expressed genes in the three comparisons. The red box indicates the up-regulated genes, and the blue box indicates the down-regulated genes. The numbers were listed in the respective boxes. (**B**) Venn diagram of vv12 vs WT and vv12 vs mutant comparisons. A total of 81 common genes were identified in both the comparisons. (**C**) GO enrichment of the 81 common genes triggered by *VvAGL12*. The number of genes is listed on the right-hand side of the panel. (**D**) Fold changes in cell wall-related genes in vv12 vs WT and vv12 vs mutant.

To elucidate the underlying nature of the phenotypic changes induced by *VvAGL12*, RNA-Seq analysis was conducted using ten-day-old seedlings of WT (Col-0), mutant (*agl12*), and *Vv12:*col grown on MS medium (Figure 7). The analysis revealed 272, 337, and 379 differentially expressed genes (DEGs) in the mutant vs WT, *Vv12:*col vs WT, and *Vv12:*col vs mutant, respectively (FDR < 0.05; log_2_ fold-change > 0.5) (Figure 7A). Among these, 82, 138, and 218 DEGs were up-regulated, whereas 190, 199, and 161 DEGs were down-regulated in the respective comparisons (Figure 7B). Furthermore, a comparison between the differentially expressed genes in *Vv12:*col vs WT and *Vv12:*col vs mutant identified 81 common genes, suggesting that these genes were primarily influenced by the introduction of *VvAGL12*. To gain insights into their potential functions, gene ontology (GO) analysis was conducted. This analysis revealed enrichment in biological processes related to the “response to environmental stimulus” (including responses to stress, water, stimulus, abiotic stimulus, and osmotic stimulus), “oxygen-containing compound”, “cell wall”, “external encapsulating structure”, and “cell periphery” (Figure 7C). Notably, the cell-wall pathway exhibited a higher *p*-value compared to other pathways, prompting a closer examination of cell wall-related genes, all of which were up-regulated in *Vv12:*col compared to both WT and the mutant (Figure 7D).

## 4. Discussion

Previous studies have highlighted the differences in the novel/non-functional and sub-functional aspects of MADS-box genes [29]. It has been established that *AGL12* serves as a pivotal factor in cell proliferation, thereby influencing the root development of *Arabidopsis thaliana* [17,30,31]. However, the functions of *VvAGL12* remain unclear. *VvAGL12* is a grape homolog of the *Arabidopsis AGL12* gene, which belongs to the MADS-box transcription factor family and is expressed in both the vegetative and reproductive organs (Figure 2). The findings of this study were partially aligned with those of previous studies. Earlier investigations revealed that *VvMADS48*, also known as *VvAGL12*, exhibits highest expression in the roots of vegetative organs, followed by fruits [32]. Conversely, this study identified that *VvAGL12* was most highly expressed in young leaves and inflorescences, followed by fruits and roots. These discrepancies may be attributed to varietal and age-related differences. Notably, Wang et al. (2014) collected samples from two-year-old “Kyoho” (*Vitis vinifera × Vitis labrusca*) and “Thompson Seedless” (*Vitis vinifera*) seedlings, whereas our study utilized samples from four-year-old “Pinot Noir” (*Vitis vinifera*) [32]. To elucidate the biological function of *VvAGL12* in plant growth and development, we isolated this gene from grapes, which exhibited substantial similarity to other MADS-box members in plants (Figure 1). In grapes, VvMADS28, VvMADS39, VvAGL11, VvAG2, and VvSEP3 have been reported to localize within the nucleus, whereas rice OsMADS25 is distributed throughout the cell [8,33,34,35]. Our observations indicated that VvAGL12 was localized in the nucleus, suggesting a potential role within this cellular compartment, akin to other nucleus-localized MADS-box proteins (Figure 2A).

Numerous studies have explored the role of MADS-box genes in plant growth and development. For instance, in *Arabidopsis*, overexpression of lavender *AGAMOUS-like* and *SEPALLATA3-like* genes has been found to accelerate flowering and alter leaf morphology [36]. In rice, *OsMADS25* has been identified as a regulator of root system development, influencing auxin signaling and nitrate accumulation [35]. Notably, previous investigations have elucidated the functions of *AtAGL12*, a gene expressed in roots, leaves, and floral meristems. It plays a pivotal role in root development by governing the transition between cell proliferation and differentiation, stem cell proliferation, and flowering [16,17]. Meanwhile, *OsMADS26*, a member of the AGL12 group, exhibited diverse phenotypes in different genetic backgrounds [18,37]. For instance, Lee et al. (2008) reported that overexpression of *OsMADS26* in japonica “Dongjin” resulted in significant defects in root and shoot growth, along with stress-related phenotypes such as chlorosis, cell death, and pigment accumulation [18]. However, in japonica “Nipponbare”, down-regulated *OsMADS26* plants displayed enhanced resistance to pathogens and improved drought tolerance with minimal effects on overall plant development [37].

To explore the potential roles of AGL12 group transcription factors in the growth and flowering time of both grapes and *Arabidopsis*, *VvAGL12* was introduced into wild-type and *agl12* mutant *Arabidopsis* plants. The generated transgenic *Arabidopsis* lines displayed varying levels of *VvAGL12* overexpression (Figure S1). Compared to wild-type, the *agl12* mutant exhibited reduced fresh weight, root length, and delayed flowering, which was partially similar to previously reported *xal1-1* and *xal1-2* alleles [17]. However, these phenotypic traits were restored in *VvAGL12* complementary lines (Figure 3, Figure 4 and Figure 5). In contrast, the overexpression of *VvAGL12* significantly accelerated plant growth and flowering, leading to increased root length, plant height, biomass, and shortened time to bolting and flowering compared to wild-type plants (Figure 3, Figure 4 and Figure 5). These observations were partially similar to those found in *Arabidopsis AtAGL12* mutants, with our study emphasizing the phenotypes across the entire growth period.

Besides its influence on plant growth and flowering time, we delved into the role of *VvAGL12* in seed production in transgenic *Arabidopsis* (Figure 7). This phenotype was first reported in the AGL12 group but not specifically within the MADS-box family. Previous studies have shown that the *Arabidopsis* MADS-domain transcription factor SEEDSTICK played a crucial role in regulating seed size through direct activation of *E2Fa*. Additionally, *PfMADS16* from *Polypogon fugax* has been identified as a factor that leads to seed abortion [38,39].

To elucidate the impact of *VvAGL12* overexpression on the growth of transgenic plants, we performed transcriptomic analyses, which revealed a substantial number of genes displaying altered expression patterns in transgenic plants and *agl12* mutants (Figure 7A–C). Further investigation revealed significant changes in genes associated with the cell wall and cell proliferation in both transgenic plants and *agl12* mutants (Figure 7D). Previous studies have established the critical role of cell-wall- and cell-proliferation-related genes in the regulation of plant growth. Xyloglucan transferases/hydrolases (XTHs) were key players in cell-wall loosening, synthesis, and restructuring, facilitating cell expansion and influencing the growth of roots, stems, and fruits [40,41,42,43]. Elevated expression of *AtXTH25* controls cellular conditions by regulating Ca^2+^ ion activity in the cell wall, resulting in accelerated hypocotyl elongation and overall plant growth [44]. Ca^2+^ ions possess a wide range of functions in plants, serving as osmolytes, membrane stabilizers, and cell wall fortifiers. Maintaining proper Ca^2+^ ion levels is crucial for normal plant growth and development [45,46,47,48]. AT3G26380 encodes a glycoside hydrolase family member that functions as a β-l-arabinopyranosidase (APSE). An *Arabidopsis apse-1* mutant displayed reduced levels of β-l-arapyranoisdase in cell walls, leading to diminished hypocotyl growth [49]. Peroxidases (PRXs) aid in lignin polymerization by oxidizing lignin monomers, and class III peroxidases affect cell-wall structure or cell elongation through direct or indirect mechanisms [50]. In *Arabidopsis*, PRX33, a cell-wall-localized peroxidase, influences cell-wall-related cell growth responses and root length [51]. The *BODYGUARD* (*BDG*) gene encodes an extracellular protein expressed in the epidermis that functions as an α-β hydrolase necessary for normal cuticle formation. The *bdg* mutant plants exhibit dwarfism, abnormal leaves, collapsed cells, and reduced trichome numbers, highlighting the role of the cuticular layer of the cell wall in growth regulation [52]. In this study, up-regulation of cell-wall-related genes (*ECS1*, *F8L10.6*, *XTH25*, *PER33*, *PER42*, *APSE*, and *BDG2*) in transgenic plants likely contributed to the enhanced growth observed in these plants.

Light, a pivotal environmental factor, exerts profound effects on plant growth and development across life cycles. The B-BOX DOMAIN PROTEIN (BBXs) family plays a central role in governing light signaling and plant photomorphogenesis, thereby orchestrating various cellular and developmental processes in plants [53,54]. The CONSTANS (CO)-FLOWERING LOCUS T (FT) regulatory module serves as a conserved mechanism for the photoperiodic control of flowering in numerous plant species [55,56]. Many BBXs interact with CO through their B-box domains to modulate their transcriptional activity, with previous research revealing that *BBX7* represses *FT* and *CO* expression, whereas *BBX24* enhances *FT* transcription [54]. In *Arabidopsis*, BBX14 interacts with CO within the nucleus and its expression curtails *CO*-mediated *FT* transcription, resulting in a delayed flowering phenotype upon overexpression and an early flowering phenotype upon down-regulation [57]. This study revealed significant down-regulation of *BBX7*, *BBX14*, and *BBX27* expression, coupled with a marked up-regulation of *BBX24* in transgenic plants (Figure S3). Prior investigations have elucidated the role of *SPL2* in promoting floral induction, conferring floral meristem identity, and causing delayed vegetative phase transitions and flowering in its mutants [58]. Here, we observed significant up-regulation of *SPL2*, suggesting its potential involvement in the early flowering of transgenic plants.

In summary, *VvAGL12* demonstrated its capacity to expedite early flowering, enhance plant growth, and bolster production by modulating the expression of genes associated with cell-wall formation and flowering in *Arabidopsis*. This finding holds significant implications for gaining deeper insights into the biological functions of *VvAGL12* in grapes.

## 5. Conclusions

The constant expression of *VvAGL12* in *Arabidopsis* resulted in early flowering, enhanced plant growth, and increased production. Early flowering was associated with changes in the expression of flowering genes, whereas accelerated growth and seed production were linked to variations in cell proliferation genes. These results offer valuable insights into the role of *VvAGL12* in the growth and development of horticultural crops.

## Data Availability

All data contained within the article.

## Supplementary Materials

The following supporting information can be downloaded at https://www.mdpi.com/article/10.3390/genes14112078/s1: Figure S1: Molecular confirmation of *VvAGL12* transgenic *Arabidopsis*. Figure S2: Phenotype of rosette leaves in four lines (Col-0, *agl12*, *Vv12:cs*, and *Vv12:*col) at reproductive growth stage. Figure S3: The fold changes in flowering genes induced by introducing *VvAGL12* in wild-type plants. Figure S4: Validation of transcriptome data using real-time quantitative PCR.
