# Supplementary material for: Ectopic Expression of MADS-Box Transcription Factor VvAGL12 from Grape Promotes Early Flowering, Plant Growth, and Production by Regulating Cell-Wall Architecture in Arabidopsis
Source: Genes (Basel). 2023 Nov 15;14(11):2078. doi: 10.3390/genes14112078 (PMC10671436; doi:10.3390/genes14112078)
Supplement: Supplementary file 1 [file genes-14-02078-s001.zip › genes-2686022-supplementary.pdf]

genes

MDPI

Article

Ectopic Expression of MADS-Box Transcription Factor *VvAGL12* from Grape Promotes Early Flowering, Plant Growth, and Production by Regulating Cell-Wall Architecture in *Arabidopsis*

Tingting Mao [1,2,†], Xueting Wang [1,2,†], Hongsheng Gao [1,2], Zijian Gong [1,2], Ruichao Liu [1,2], Ning Jiang [1,2], Yaru Zhang [1,2], Hongxia Zhang [1,2,3], Xiaotong Guo [1,2] and Chunyan Yu [1,2,*]

Supplementary Materials:

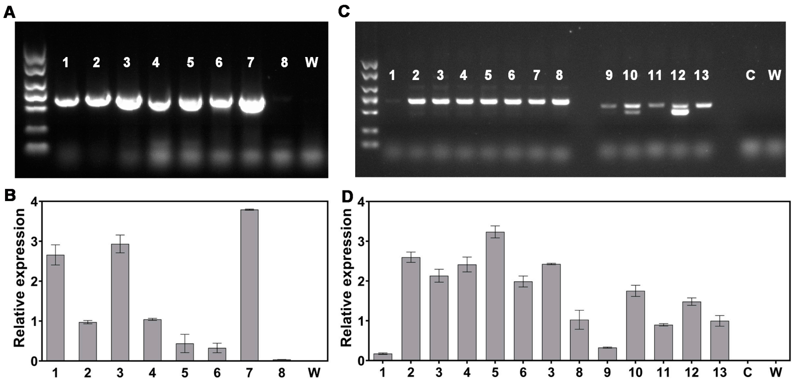

Figure S1: Molecular confirmation of *VvAGL12* transgenic *Arabidopsis*. (A) Identification of 8 overexpression of *VvAGL12* lines via PCR. (B) Evaluation of expression levels in eight *VvAGL12* overexpressing lines. (C) Identification of 13 lines with complementary mutant *agl12* by PCR. (D) Evaluation of expression levels in 13 complementary lines. The numbers represent the transgenic lines, W represents the wild-type, and C represents the water control.

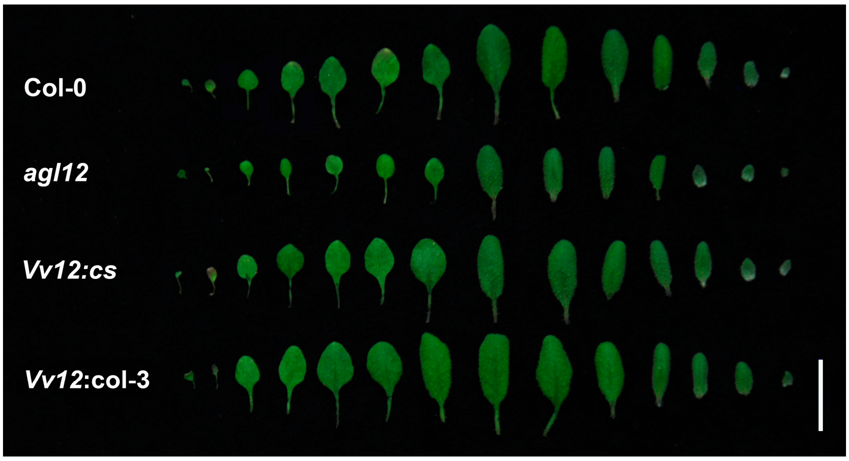

Figure S2: Phenotype of rosette leaves in four lines (Col-0, *agl12*, *Vv12:cs*, and *Vv12*:col) at reproductive growth stage. Col-0 was a wild-type *Arabidopsis*, *agl12* was a mutant of AGL12, *Vv12: cs* was the *VvAGL12* complementary *Arabidopsis* mutant *agl12*, and *Vv12*: col was overexpressed *VvAGL12* in the wild-type.

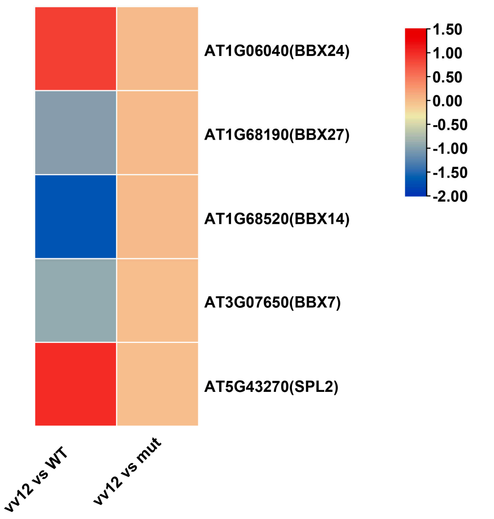

Figure S3: The Fold changes in flowering genes induced by introducing *VvAGL12* in wild-type plants.

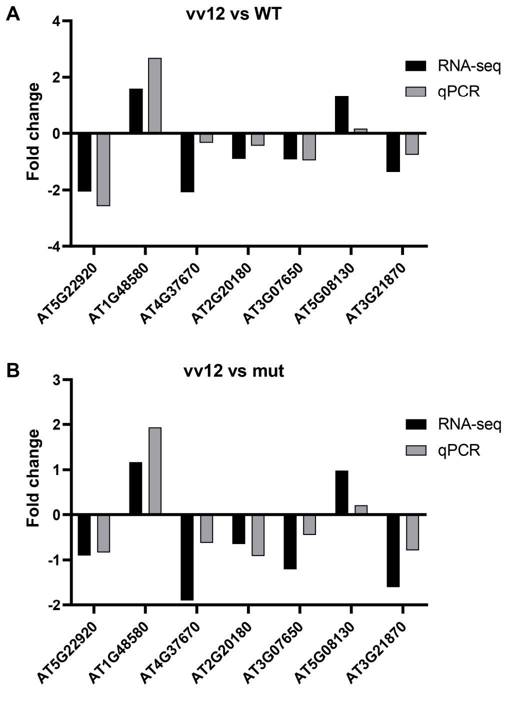

Figure S4: Validation of transcriptome data using real-time quantitative PCR. (A) Comparison between transcriptome and real-time quantitative PCR data in vv12 vs WT. (B) Comparison between transcriptome and real-time quantitative PCR data in vv12 vs Mut.
